# Research on the electromyography-based pattern recognition for inter-limb coordination in human crawling motion

**DOI:** 10.3389/fnins.2024.1349347

**Published:** 2024-03-14

**Authors:** Chengxiang Li, Xiang Chen, Xu Zhang, De Wu

**Affiliations:** ^1^School of Information Science and Technology, University of Science and Technology of China (USTC), Hefei, Anhui, China; ^2^Department of Pediatrics, The First Affiliated Hospital of Anhui Medical University, Hefei, Anhui, China

**Keywords:** pattern recognition, hands-knees crawling, electromyography, inter-limb coordination, BiLSTM

## Abstract

Aiming to provide a feasible crawling motion analysis method for clinical application, this study introduced electromyography (EMG)-based motion intention recognition technology into the pattern recognition of inter-limb coordination during human crawling for the first time. Eight inter-limb coordination modes (ILCMs) were defined. Ten adult participants were recruited, and each participant performed hands-knees crawling at low, medium, and fast speeds in self-selected ILCMs and the eight predefined ILCMs, respectively. EMG signals for pattern recognition were collected from 30 limbs and trunk muscles, and pressure signals for crawling cycle segmentation were collected from the left palm. The pattern recognition experiments were conducted in participant-specific, multi-participant, and participant-independent ways, respectively, adopting three different classifiers, including bidirectional long short-term memory (BiLSTM) network, support vector machine (SVM), and k-nearest neighbor (KNN). The experimental results show that EMG-based pattern recognition schemes could classify the eight ILCMs with high recognition rates, thereby confirming the feasibility of providing an EMG-based crawling motion analysis method for clinical doctors. Furthermore, based on the classification results of self-selected ILCMs at different speeds and the statistical results of stance duration, swing duration, and the duty factors of stance phase, the possible reasons why humans chose various ILCMs at different crawling speeds were discussed. The research results have potential application value for evaluating crawling function, understanding abnormal crawling control mechanisms, and designing rehabilitation robots.

## 1 Introduction

The acquisition of crawling skills is regarded as one of the most crucial developmental milestones in human motor skill development (Chen et al., 2017; Xiong et al., 2021). Various physical abilities can be promoted by crawling movement in infancy, including eye-hand coordination, balance, and spatial concepts (McEwan et al., 1991). Therefore, the evaluation of the crawling function has great application value in the fields of disease diagnosis and rehabilitation treatment (Gao et al., 2018; Xiong et al., 2018a,b, 2021). In particular, as a typical quadruped movement (Cole et al., 2019), there are various kinds of inter-limb coordination modes (ILCMs) during crawling. Figuring out the ILCMs during crawling may help clinicians better evaluate patients’ motor dysfunction and then develop more precise rehabilitation treatment plans.

Early studies on ILCMs during crawling were mainly based on the subjective observation and judgment of observers (Hildebrand, 1967; Freedland and Bertenthal, 1994). In this century, various kinetics-based sensing technologies, i.e., motion capture technology (Patrick et al., 2009, 2012; MacLellan et al., 2012, 2013, 2017; Righetti et al., 2015; Gao et al., 2018; Xiong et al., 2018a,b), inertial measurement unit (IMU) (Vitali et al., 2019), and 3-axis accelerometers (Ma et al., 2017), have been successfully introduced into motion analysis. Patrick et al. (2009) defined ipsilateral phase lag (IPL) value to quantify ILCMs during human crawling. In their definition, when *a* represented the whole crawling cycle and *b* represented the phase difference between the moment when the left palm and the left leg contacted the ground, IPL can be calculated as (*b*/*a*) * 100%. IPL values closed to 50% indicated trot gait, where diagonal limbs moved in coordination; IPL values closed to 0 or 100% indicated pace gait, where ipsilateral limbs moved in coordination; IPL values closed to 25% or 75% indicated no-limb-pairing gait, where all limbs moved at regular intervals (Patrick et al., 2009). Using a motion capture system, Patrick et al. carried out research on the ILCMs during human crawling and stated that infants would like to adopt trot gait (Patrick et al., 2009). Using 3-axis accelerometers and pressure sensors, Ma et al. investigated hands-knees crawling in adult humans and found that, at low speeds, most adults crawled using trot or no-limb-pairing gait, while they tended to use trot or pace gait as their crawling speed increased (Ma et al., 2017).

In summary, current research on ILCMs was in the preliminary stage, and the research results had certain limitations. First, crawling is a full-body movement related to the coordinated contraction of a series of limbs and trunk muscles. It is universally acknowledged that motion capture systems have high light requirements in the experimental environment, which is usually a fixed experimental site and is easily affected by occlusion, resulting in data loss. Inertial sensors such as IMU and ACC will produce cumulative errors, affecting the accuracy of analysis. Specifically, neither subjective observation nor kinetics-based sensing technologies can reflect the motor control characteristics of the central nervous system (CNS) from the perspective of muscle contraction. Second, most of the existing studies divided the ILCMs into trot gait, pace gait, and no-limb pairing gait according to the IPL value (Patrick et al., 2009, 2012; MacLellan et al., 2012, 2013; Righetti et al., 2015; Chen et al., 2017; Ma et al., 2017; Vitali et al., 2019). However, the IPL was difficult to distinguish modes with subtle differences. For instance, during hands-knees crawling, the trot gait (diagonal limbs moving together) had the same IPL value (50%) as sequential gait (left palm → right knee → left knee → right palm). Third, although relevant studies have found that human beings have different choices for the ILCMs at different crawling speeds (Chen et al., 2017; Ma et al., 2017), there was a lack of exploration of the possible reasons for making the choice.

In view of the shortcomings of existing research, this study attempted to carry out research on the pattern recognition of ILCMs during crawling by means of electromyography (EMG) signals, which carry abundant muscle activation information and neuromuscular control information of the CNS. Compared to motion capture systems and inertial sensors, EMG signals do not require a specific experimental site and are not affected by cumulative errors. Therefore, it has been widely used for pattern recognition of human motion intention, such as ankle joint movements (Al-Quraishi et al., 2017), lower limb jump locomotion phases (Lu et al., 2021), hand gestures (Côté-Allard et al., 2019), and muscle forces (Mokri et al., 2022). The following benefits can be achieved by introducing EMG-based motion intention recognition technology into the inter-limb coordination pattern recognition. First, given the successful application of EMG in fine finger motion recognition (Côté-Allard et al., 2019), we believe that it can achieve better accuracy in coarse crawling motion recognition; Second, unlike roughly dividing the ILCMs into trot, pace, and no-limb-pairing gait, using EMG signals recorded from related muscles was expected to achieve more accurate classification; Third, the pattern recognition scheme for ILCMs based on EMG signals can provide a novel crawling motion analysis technology, which was helpful for clinicians to evaluate patients’ motor dysfunction status from the perspective of muscle function and also helped researchers understand the neuromuscular control mechanism during human crawling.

The fundamental purposes of this study are as follows: (1) first introduce EMG-based motion intention recognition technology into crawling motion classification; (2) explore the reasons for the choice of different ILCMs at various crawling speeds based on the classification results and kinetic parameters of self-selected ILCM under the pattern recognition scheme. The innovations and primary contributions can be summarized as follows: (1) Unlike relevant research studies, which roughly divided the crawling modes into three gaits, this study targeted eight ILCMs defined by the sequence in which limbs contact the ground; (2) The feasibility of providing clinicians with an EMG-based ILCM pattern recognition scheme has been verified by pattern recognition experiments in participant-specific way, multi-participant way, and participant-independent way respectively, with three classifiers including BiLSTM, SVM, and KNN; (3) By classifying the participants’ self-selected ILCM at different speeds using the best classification model, the possible reasons why the choice of the ILCM changed with crawling speed were discussed.

## 2 Materials and methodology

**Figure 1:**
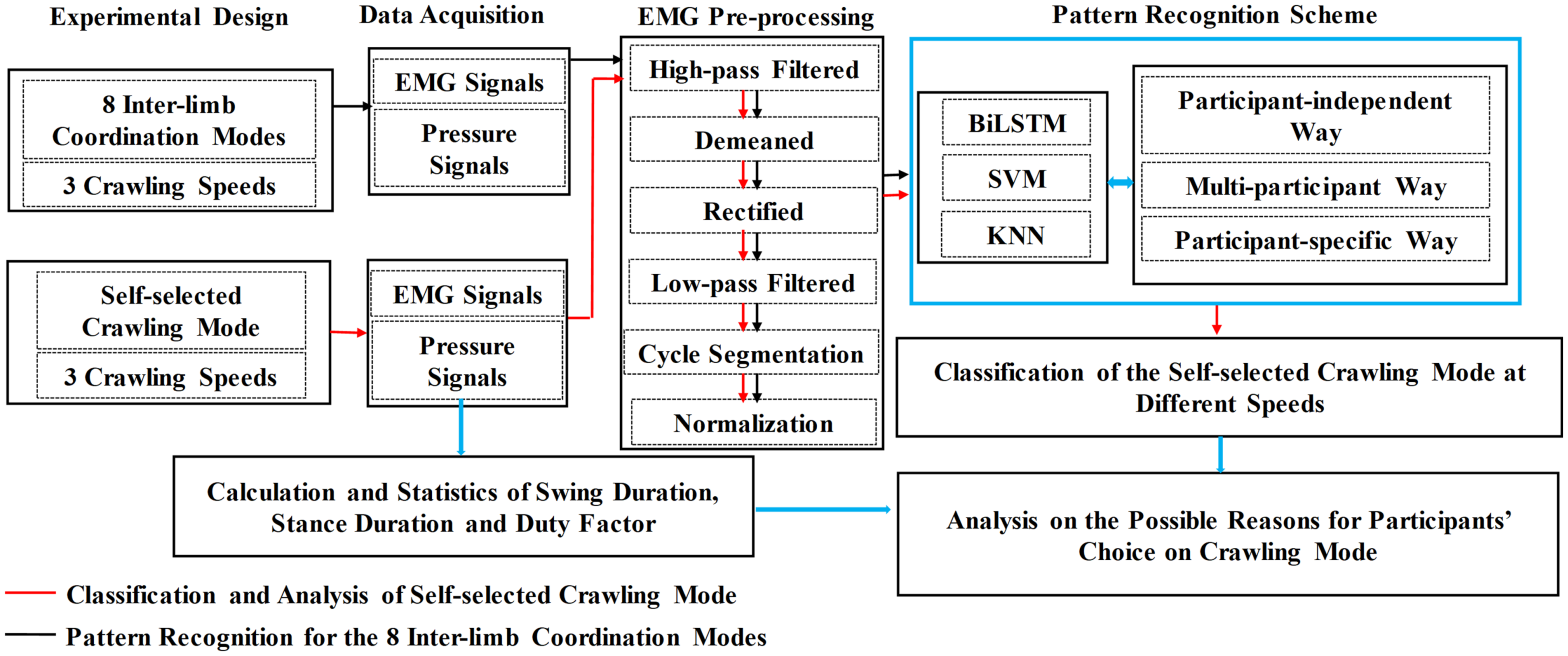
Flowchart of the research route.

The research flowchart of this study is illustrated in Figure 1, and each step is described in detail below.

### 2.1 The experimental design and crawling data acquisition

Eight ILCMs were defined, as shown in Table 1. Each crawling cycle was initiated by the left palm touchdown and defined as the time interval between two consecutive left palm touchdowns (Patrick et al., 2009; MacLellan et al., 2012; Chen et al., 2017).

**Table 1:** The eight designed inter-limb coordination modes.

Modes	Ideal IPL value	Order of limbs touching land
M1	0(100%)	LP, LK → RP, RK
M2	50%	LP, RK → RP, LK
M3	50%	LP → RP → LK → RK
M4	75%	LP → RP → RK → LK
M5	25%	LP → LK → RP → RK
M6	25%	LP → LK → RK → RP
M7	75%	LP → RK → RP → LK
M8	50%	LP → RK → LK → RP

LP, left palm; RP, right palm; LK, left knee; RK, right knee.

The crawling data acquisition experiment involved the participation of 10 healthy adults, comprising three female subjects and seven male subjects, with an average age of 23.90 ± 0.88 years. None of the participants had a previous diagnosis of neuromuscular disorders. This study was approved by the Ethics Review Committee of Anhui Medical University (No. PJ 2014-08-04). All participants were informed about the study details and provided written informed consent.

**Figure 2:**
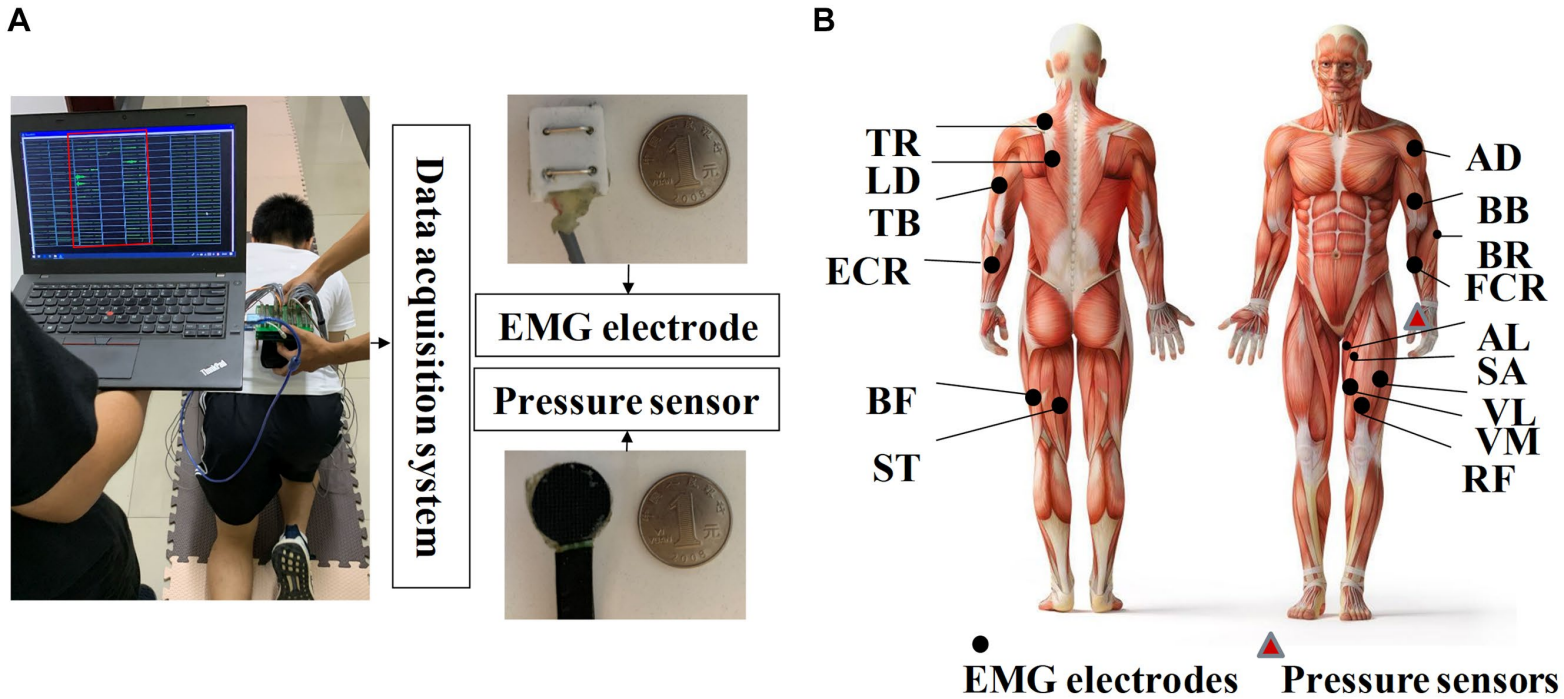
**(A)** Illustration of the homemade data acquisition system; **(B)** placement of EMG sensors and pressure sensors.

A laboratory-made multi-channel system with 30 EMG sensors and 1 pressure sensor was used to collect crawling data. As shown in Figure 2A, EMG electrodes contained bipolar separating silver wires 23 mm in length and 20 mm in width, with a 10-mm interval between them (Li et al., 2023). As shown in Figure 2B, EMG signals were recorded from 15 muscles on each side of the body and 30 muscles from the whole body in total, which were highly related to crawling, including anterior deltoid (AD), adductor longus (AL), biceps brachii (BB), biceps femoris (BF), brachioradialis (BR), extensor carpi radialis (ECR), flexor carpi radialis (FCR), latissimus dorsi (LD), rectus femoris (RF), sartorius (SA), semitendinosus (SE), triceps brachii (TB), trapezius (TR), vastus lateralis (VL), and vastus medialis (VM). The placement of EMG sensors was based on the guidelines of the SENIAM protocol (Hermens et al., 2000). Before placing the sensors, the target muscles were shaved and cleaned with alcohol swabs. To detect crawling cycles, a pressure sensor was affixed with kinesiology tape to the flexor pollicis brevis muscle of the left palm. The company WAAAX manufactured the RP-C18.3-ST thin-film piezoresistive pressure sensor, which has a diameter of 18.3 mm. The sampling rate of EMG electrodes and pressure sensor was set to 1,000 Hz.

Throughout the data acquisition experiment, participants crawled on a sponge pad measuring 11.2 m in length and 0.8 m in width. First, each participant crawled in self-selected ILCM at their self-understanding slow, medium, and fast speeds. From the pressure signal on the left palm of the participants, the low, medium, and fast speeds in the self-selected ILCM were calculated to be approximately 2 s/cycle, 1.5 s/cycle, and 1 s/cycle, respectively. Then, each participant was asked to crawl in the eight ILCMs (M1 to M8) at three different speeds, namely slow speed (approximately 3.5 s/cycle), medium speed (approximately 2.33 s/cycle), and fast speed (approximately 1.75 s/cycle). To help participants complete crawling movements at specific speeds and ILCMs, a series of audio files prompting the landing order of each limb was generated for the eight ILCMs at three crawling speeds. Before data collection, the participants learned how to crawl under the alert of audio files until they became proficient. For any combination of speed (fast, medium, and slow) and ILCM (M1 to M8, self-selected), as a crawling trial, pressure signals and EMG signals for at least 15 consecutive crawling cycles were collected. To minimize muscle fatigue, participants were required to rest for about 10 min between each group of 6 crawling trials. Table 2 displays the total number of crawling cycles for each participant across all eight ILCMs (M1–M8) and their self-selected crawling mode.

**Table 2:** Number of crawling cycles.

Crawling mode	Participant ID	P1	P2	P3	P4	P5	P6	P7	P8	P9	P10
M1–M8	Low speed	95	66	85	93	81	92	86	88	104	109
	Medium speed	105	77	90	96	85	95	98	90	91	109
	Fast speed	102	63	96	97	85	86	91	82	87	112
Self-selected crawling mode	Low speed	10	9	8	9	8	10	9	10	10	11
	Medium speed	11	11	10	10	8	9	9	10	11	10
	Fast speed	10	8	10	9	10	9	9	10	10	10

It is worth noting that this study proposed the following countermeasures for the artifacts that may be introduced by data collection systems in different scenarios: (1) To avoid power frequency noise, lithium battery was used to power the data acquisition system; (2) The experimental operator checked the interface connection between each electrode and the data acquisition system to ensure that all sensor connections were normal before the experiment begins; (3) The sensor connecting lines located on the same limb were fixed together with adhesive tape to reduce the disturbance; (4) During crawling data collection, the experimental operator observed the real-time signal on the laptop and independently stored the data of each crawling mode. Once the artifact was discovered, the experiment was suspended, and the current experimental data were discarded. Only after the cause of the artifact was found and the fault was eliminated can the experiment be restarted.

### 2.2 Data preprocessing

**Figure 3:**
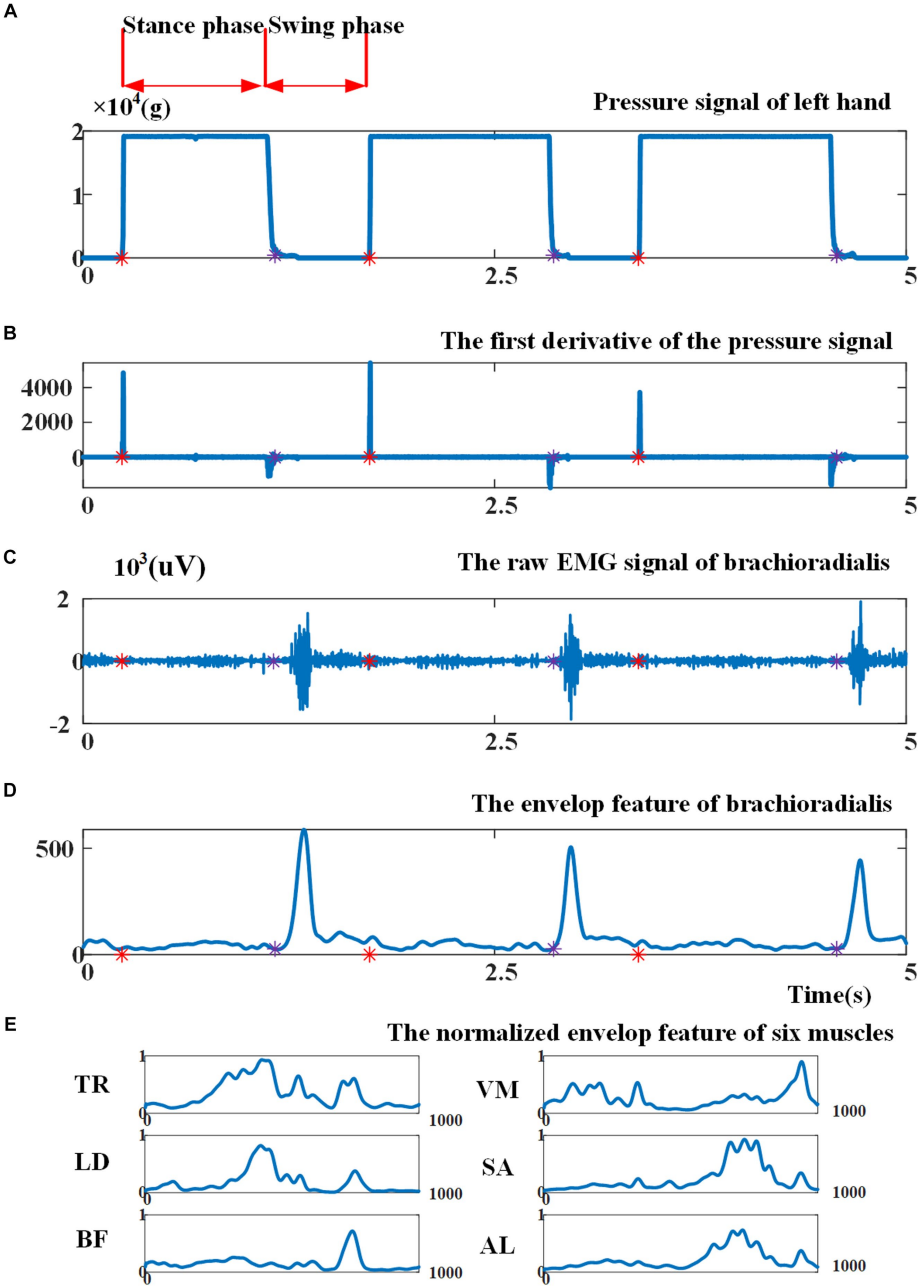
**(A)** Illustration of the pressure signal collected from left palm; **(B)** the first derivative of the pressure signal. Red asterisks indicate cycle starting points, as the beginning point of the stance phase, and purple asterisks indicate the beginning point of the swing phase; **(C)** illustration of raw EMG signal from brachioradialis; **(D)** the envelope of EMG signal before cycle normalization and amplitude normalization; **(E)** the envelope of EMG signal of six muscles after cycle normalization and amplitude normalization.

Figure 3A illustrates the pressure signal obtained during a consecutive crawling movement. When the left palm touched the land, the left palm entered into the stance phase and the pressure signal reached the maximum value; when the left palm left the land, the left palm entered into the swing phase and the pressure signal returned to zero. Figure 3B shows that the first derivative of the pressure signal during left-palm stance and swing phase was zero. When transitioning from the swing phase to the stance phase, the first derivative became greater than zero; when transitioning from the stance phase to the swing phase, it became less than zero. Therefore, this study utilized the aforementioned characteristics of the pressure signal’s first derivative to segment crawling cycles. In Figure 3, the red asterisks indicated crawling cycle starting points and the purple asterisks indicated swing phase starting points. The crawling cycle was segmented by two adjacent red asterisks.

Various EMG features can be extracted for EMG-based motion intention pattern recognition, including (1) time-domain features, such as mean absolute value, slope sign changes, and waveform length (Hudgins et al., 1993); (2) frequency-domain features based on fast Fourier transform, i.e., intermediate frequency (MDF), average frequency (MNF), autoregressive coefficient (AR), etc. (Abdelouahad et al., 2018); and (3) time-frequency-domain features based on Wigner-Ville transform (WVT), wavelet transform, etc. (Li et al., 2020). Given that the variation in muscle activation intensity can indicate the differences between ILCMs, this study opted to utilize an EMG signal envelope as a feature for pattern recognition. In particular, the EMG signals of each crawling cycle were high-pass filtered, demeaned, rectified, and low-pass filtered to extract the envelope (Li et al., 2023). Then, the envelope amplitude was normalized to unit variance (Teruya et al., 2021), and each crawling cycle length was normalized to 1,000 points. As an example, Figures 3C, D present the original EMG signal fragment from the brachioradialis and its envelope, respectively, before cycle normalization and amplitude normalization. Figure 3E demonstrates the EMG signal envelopes of six muscles after undergoing cycle normalization and amplitude normalization.

### 2.3 EMG-based crawling pattern recognition scheme

To verify the feasibility of providing clinicians with an EMG-based scheme for accurate recognition of ILCM, the pattern recognition experiments on the eight defined ILCMs were carried out in the participant-specific way, multi-participant way, and participant-independent way, respectively, at four crawling speeds, including low, medium, fast, and mixed speed (EMG data mixed from low, medium, and fast speeds), with three classifiers, namely BiLSTM network, SVM, and KNN.

#### 2.3.1 Three classifiers

BiLSTM, SVM, and KNN were adopted to complete the pattern recognition task for crawling motion with different ILCMs based on the following considerations: (1) SVM model has kernel trick characteristic, KNN has non-parametric nature (Samuel et al., 2019), and these two classifiers have been widely adopted within the realm of EMG-based pattern recognition; (2) both KNN and SVM classifiers were characterized by their ease of implementation and efficient training (Samuel et al., 2019); and (3) LSTM model, which was good at memorizing the timing correlation, has been applied successfully in EMG-based gesture recognition (Chen et al., 2020). As a variation of LSTM, the BiLSTM network had better performance than the regular LSTM network. KNN and SVM were implemented by using Python’s sklearn toolkit. This section mainly introduced the implementation of the BiLSTM network.


(1) $$F_{f}=\delta \left(W_{fh}\cdot h_{t-1}+W_{fx}\cdot x_{t}+b_{f}\right)$$

(2) $$F_{u}=\delta \left(W_{uh}\cdot h_{t-1}+W_{ux}\cdot x_{t}+b_{u}\right)$$

(3) $$F_{o}=\delta \left(W_{oh}\cdot h_{t-1}+W_{\mathrm{ox}}\cdot x_{t}+b_{o}\right)$$

(4) $${\bar{C}}_{t}=\tanh\left(W_{ch}\cdot h_{t-1}+W_{cx}\cdot x_{t}+b_{c}\right)$$

(5) $$C_{t}=F_{f}\cdot C_{t-1}+F_{u}\cdot {\bar{C}}_{t}$$

(6) $$h_{t}=F_{o}\cdot \tanh\left(C_{t}\right)$$

**Figure 4:**
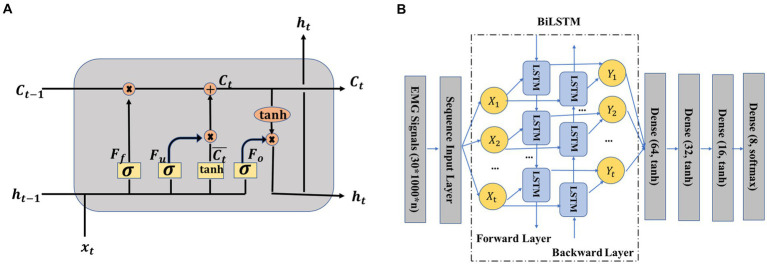
**(A)** Structure of long short-term memory (LSTM) unit (Hermens et al., 2000; Cole et al., 2019), **(B)** crawling pattern recognition based on EMG and bidirectional LSTM (BiLSTM).

To better understand how the BiLSTM network works, we first figured out its most important part, namely the LSTM unit (Choi et al., 2019; Marentakis et al., 2021). As shown in Figure 4A, an LSTM unit consisted of several main parts, including the input information $x_{t}$ at step time *t*, memory cell state $C_{t}$, temporary memory cell state ${\bar{C}}_{t}$, hidden state $h_{t}\text{,}$ and three gates (forget gate $F_{f}$, update gate $F_{u}$, and output gate $F_{o}$). The previous memory cell $C_{t-1}$, previous hidden state $h_{t-1}$, and current input $x_{t}$ decided the output hidden state $h_{t}$ and memory cell $C_{t}$ together. The temporary memory cell state ${\bar{C}}_{t}$ was decided by a tanh layer based on the previous hidden state $h_{t-1}$ and current input $x_{t}$. The functions of the three gates can be summarized as follows: the forget gate $F_{f}$ decided the information to be thrown away by a sigmoid layer, the update gate $F_{u}$ decided that the information should be stored in the next LSTM unit and the output gate $F_{o}$ decided what should be ultimately output. The specific calculation process of the LSTM unit can be seen from formula (1)–(6), where σ (·) was the sigmoid function, *W* represented weight matrices, and *b* denoted biases.

The BiLSTM network structure adopted in this study is presented in Figure 4B. The working principle of the BiLSTM network was that the input data sequence$X=\left(X_{1},X_{2},\ldots X_{t}\right)$ was not only fed to a LSTM network named the forward layer but also was simultaneously fed to another LSTM network named the backward layer by reversing the order of the input sequence of data. In other words, the first sequence $X_{1}$ was the input of the first unit of the forward layer and also the input of the last unit of the backward layer. The output of the forward layer and the output of the backward layer jointly decided the final output *Y*_1_. After the BiLSTM layer, four dense layers were adopted. Python’s Keras toolbox was adopted to implement this network. The adaptive moment estimation (Adam) was used to avoid overfitting. The hyperparameters were listed as follows: learning rate = 0.001, batch size = one-tenth of the training samples, beta_1 = 0.9, beta_2 = 0.999, epsilon = 1e-08, LSTM hidden units = 128, and training epochs = 60.

#### 2.3.2 Three pattern recognition ways

In the participant-specific way, the test data and training data were from the same participant. For each participant, a three-fold cross-validation was performed, where two-thirds of the data were used for training and the remaining one-third was used for testing.

In the multi-participant way, the data from all 10 participants were combined, and three-fold cross-validation was adopted, where two-thirds of the data were used for training and the remaining one-third was used for testing.

In the participant-independent way, the leave-one-out strategy was adopted. Classifiers were trained using crawling data obtained from nine participants, and the well-trained classifiers were applied to test the remaining crawling data.

#### 2.3.3 Performance evaluation and statistical analysis

The pattern recognition accuracy was determined by calculating the ratio of correctly identified samples to the total number of samples. To investigate the effects of the independent variables (pattern recognition way, classifier, and crawling speed) on the recognition accuracy, a one-way ANOVA and univariate ANOVA were conducted using IBM SPSS Statistics 26. The significance level was set at 0.05.

### 2.4 Analysis scheme for the possible reasons for the choice of self-selected crawling mode

As shown in Figure 1, the analysis of the possible reasons why participants’ choice of ILCM varied with crawling speed was based on the classification results of self-selected ILCM and the statistical results of the stance duration, swing duration, and duty factor of the stance phase.

The classification model, performing the best in the pattern recognition experiment on the eight defined ILCMs, was used to classify the participants’ self-selected crawling mode at different speeds. The stance phase durations and swing phase durations for the left palm were calculated according to the pressure signal, as shown in Figure 3A. The duty factor of the stance phase was calculated according to the following formula (7):


(7) $$\mathrm{Duty}\;\mathrm{factor}=\frac{\mathrm{Stance}\;\mathrm{phase}}{\mathrm{Stance}\;\mathrm{phase}+\mathrm{Swing}\;\mathrm{phase}}*100\%$$

## 3 Results

### 3.1 The pattern recognition results for the eight defined ILCMs

**Figure 5:**
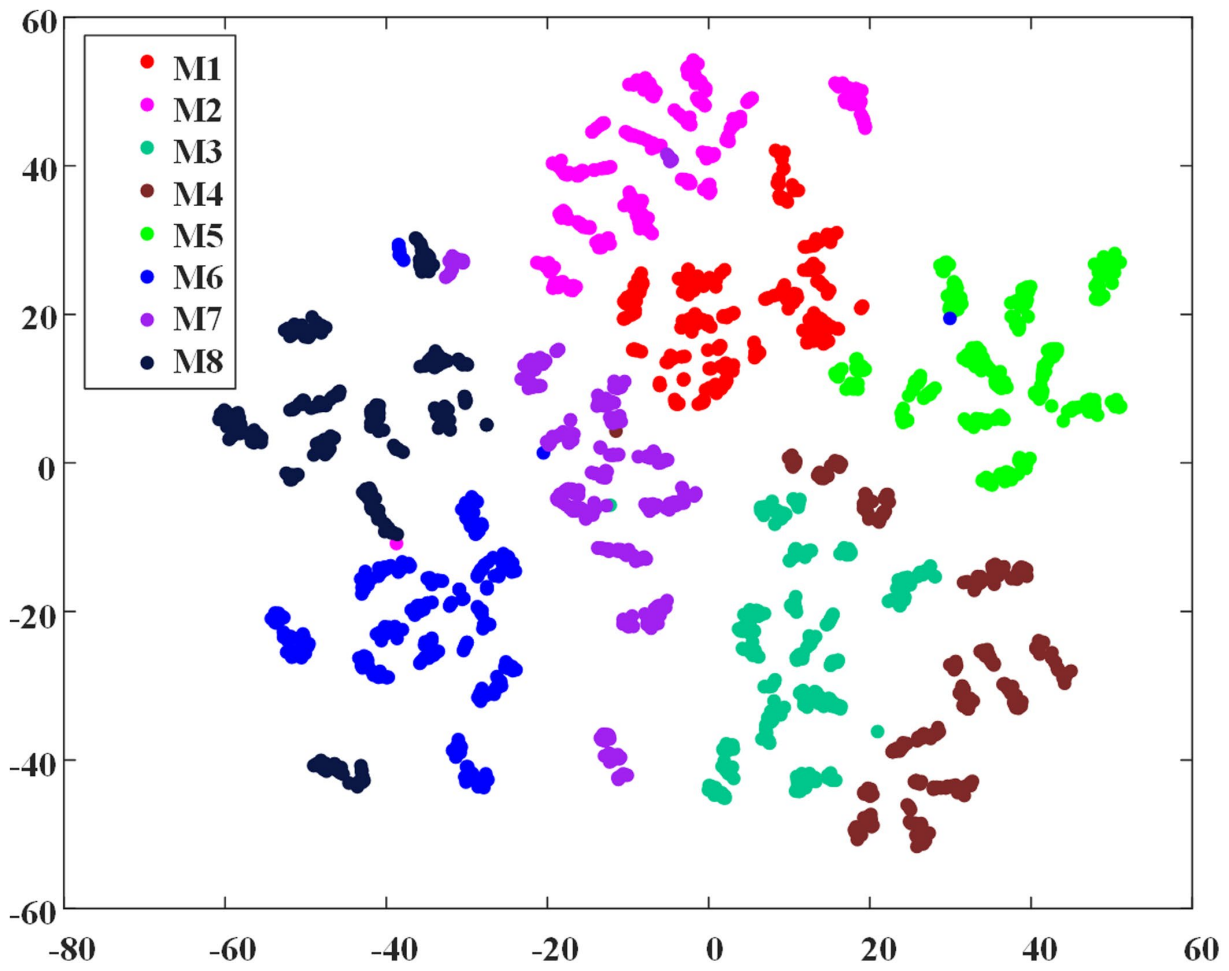
The t-SNE dimensionality reduction of EMG envelope samples of the eight inter-limb coordination modes.

To demonstrate the feasibility of the EMG envelope feature in distinguishing different ILCMs, the t-SNE dimensionality reduction algorithm (Van der Maaten and Hinton, 2008) was carried out on all the 2,736 crawling cycles of M1 ~ M8, and Figure 5 shows the visualization results. It can be observed that the eight defined ILCMs can be clearly distinguished from each other.

**Figure 6:**
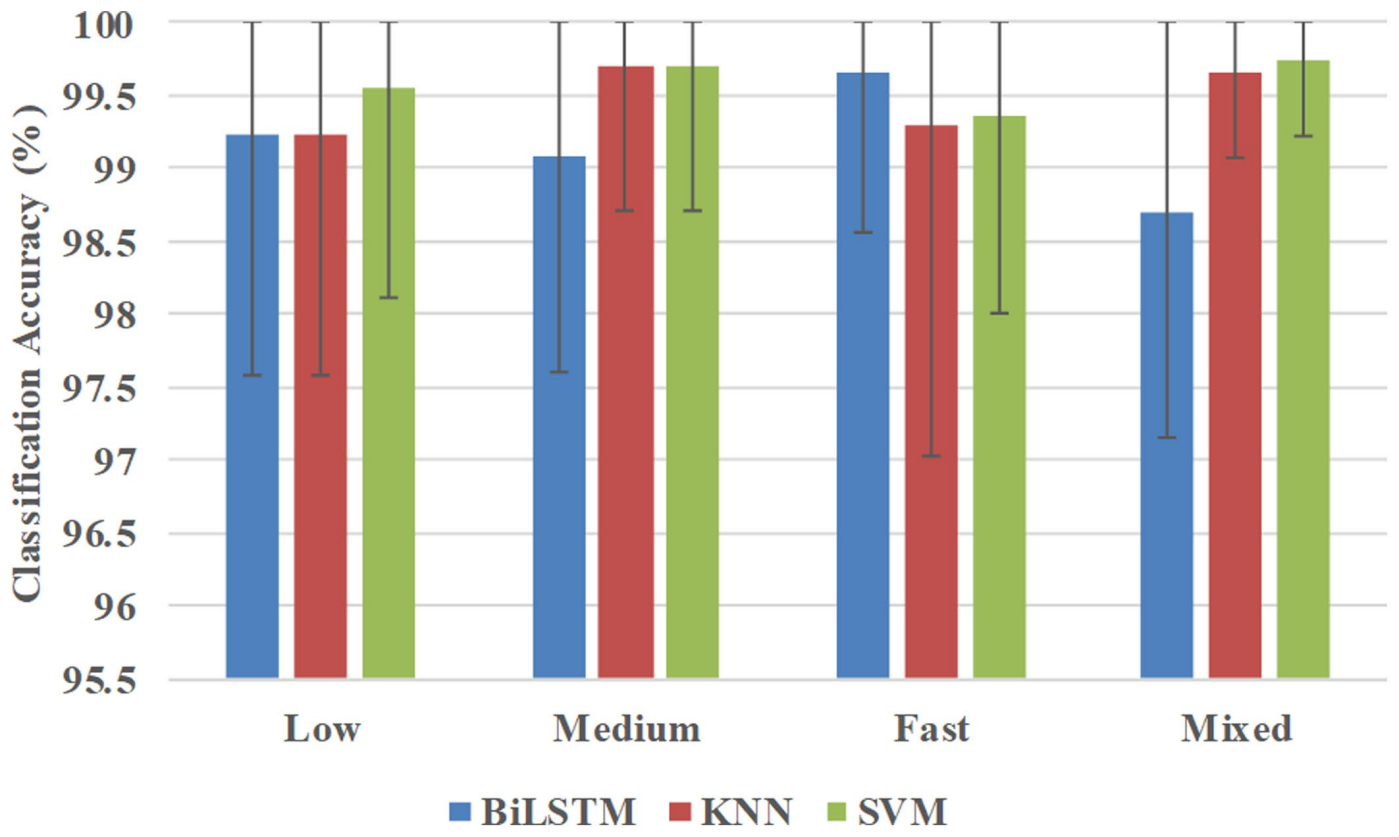
Classification accuracies were averaged across all 10 participants in the participant-specific way.

Figure 6 shows the classification accuracies averaged across all 10 participants in the participant-specific way. BiLSTM obtained average accuracies of 99.23, 99.09, 99.66, and 98.71% at low, medium, fast, and mixed speeds, respectively. KNN achieved average accuracies of 99.23, 99.69, 99.29, and 99.65% at low, medium, fast, and mixed speeds, respectively. SVM obtained average accuracies of 99.55, 99.69, 99.36, and 98.75% at low, medium, fast, and mixed speeds, respectively.

**Figure 7:**
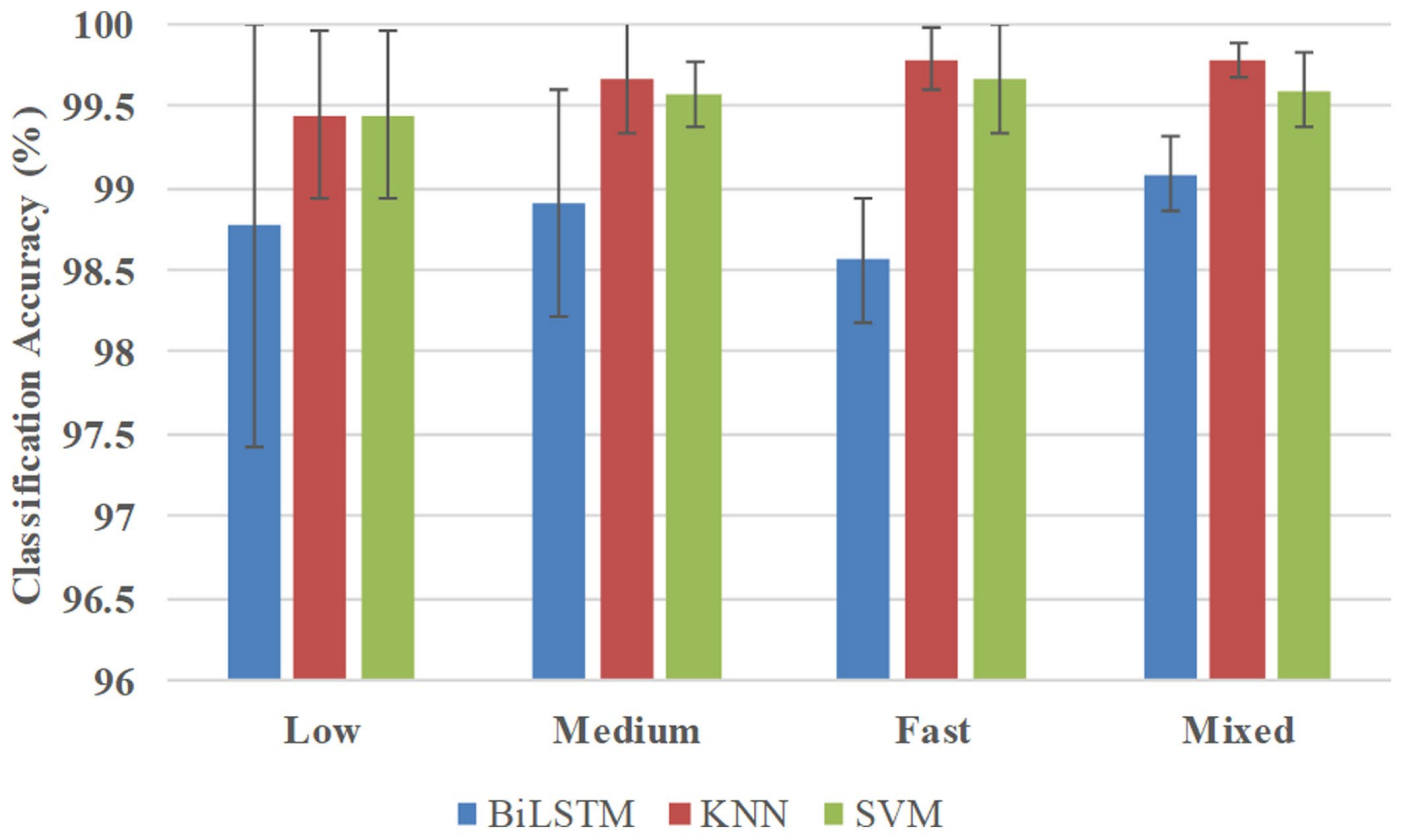
Classification accuracies in the multi-participant way.

Figure 7 shows the classification accuracies in the multi-participant way, and BiLSTM obtained the average accuracies of 98.78, 98.91, 98.56, and 99.08% at low, medium, fast, and mixed speeds, respectively. KNN obtained the average accuracies of 99.44, 99.67, 99.78, and 99.78% at low, medium, fast, and mixed speeds, respectively. SVM obtained average accuracies of 99.44, 99.56, 99.67, and 99.60% at low, medium, fast, and mixed speeds, respectively.

**Figure 8:**
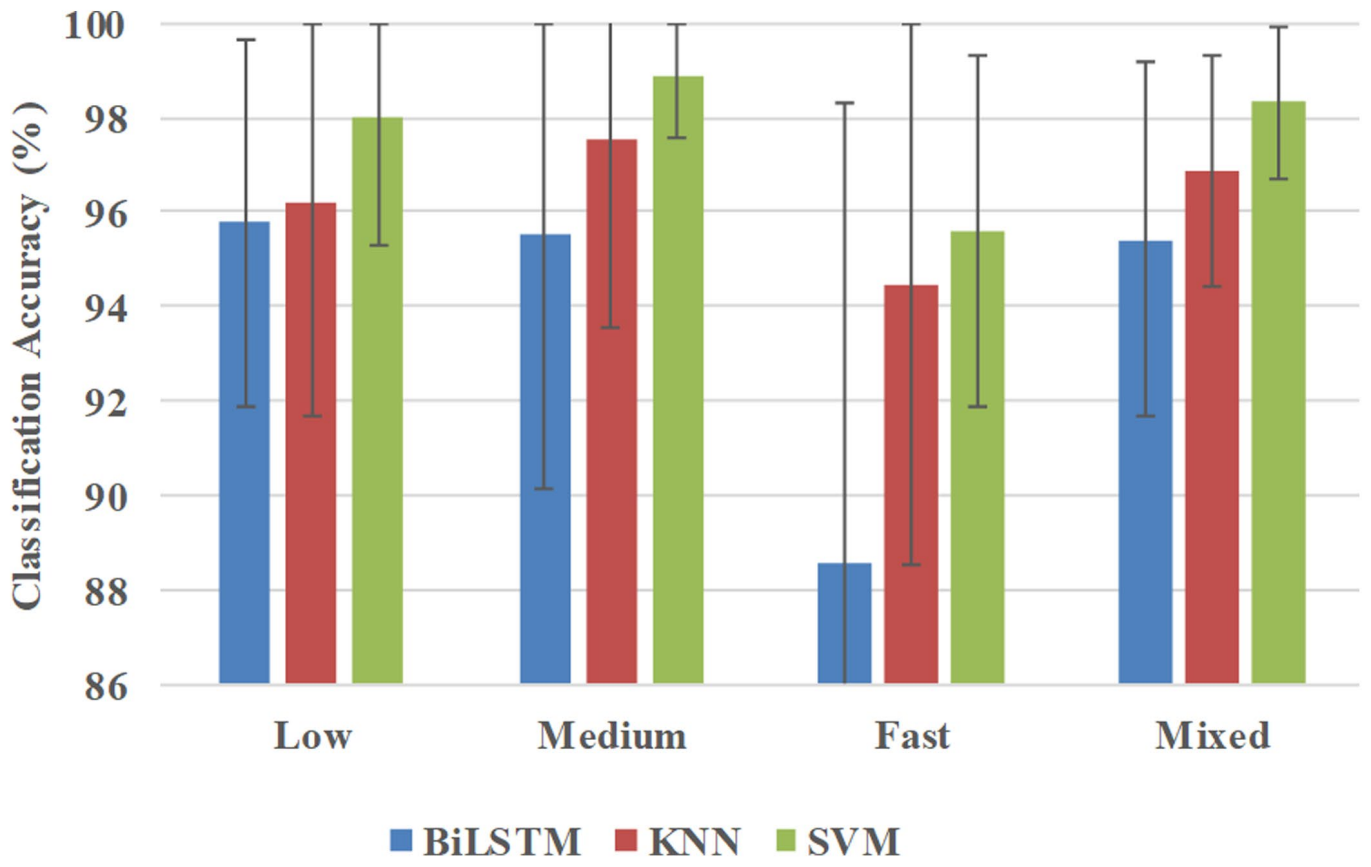
Classification accuracies were averaged across all 10 participants in the participant-independent way.

Figure 8 shows the classification accuracies in the participant-independent way, and BiLSTM obtained the average accuracies of 95.76, 95.51, 88.56, and 95.42% at low, medium, fast, and mixed speeds, respectively. KNN obtained the average accuracies of 96.21, 97.54, 94.43, and 96.89% at low, medium, fast, and mixed speeds, respectively. SVM obtained average accuracies of 98.31, 98.89, 95.60, and 98.31% at low, medium, fast, and mixed speeds, respectively.

**Figure 9:**
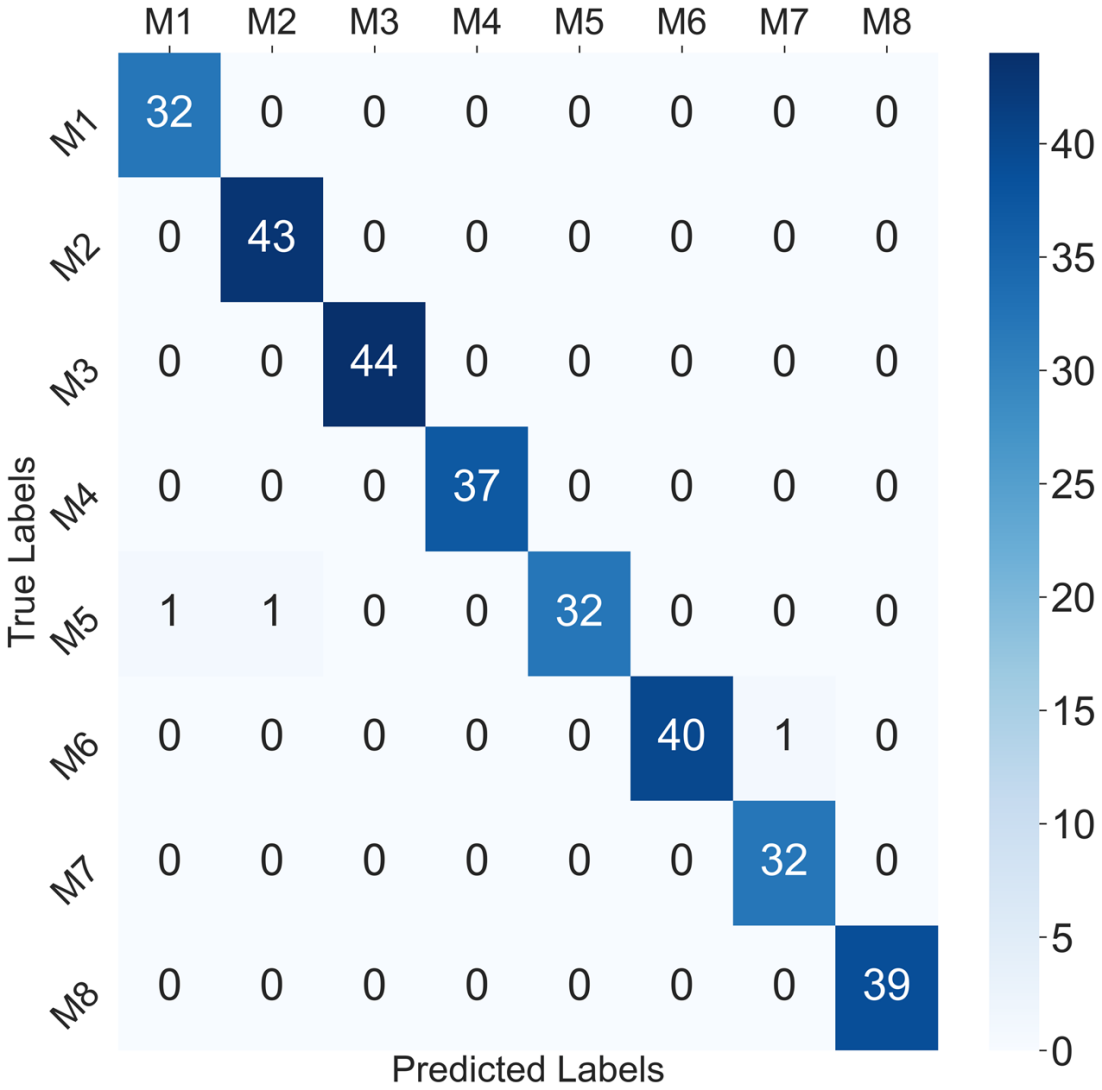
Confusion matrix for SVM classifier at mixed speed in the participant-independent way.

Considering that the SVM classifier achieved the best performance in the participant-independent way, taking the 302 crawling samples from participant P1 as test data, the confusion matrix for the SVM classifier at mixed speed was given in Figure 9. Only one sample of M1 and one sample of M2 were misclassified as M5, and one sample of M7 was misclassified as M6. The recognition accuracy of some ILCMs, such as M3, M4, M5, M6, and M8, was 100%.

Table 3 presents the results of the statistical analysis examining the effects of pattern recognition way, classifier, and crawling speed on recognition accuracy. Based on the findings, the following conclusions can be drawn: (1) Pattern recognition way had a considerable effect on the recognition accuracy. More precisely, the recognition accuracy in the participant-independent way was notably inferior to that in the participant-specific way (*p* = 0.000**) and multi-participant way (*p* = 0.000**). Nonetheless, no significant distinction was found (*p* = 0.938) between the participant-specific way and the multi-participant way; (2) Crawling speed had a significant impact on recognition accuracy. However, only fast speed had a significant difference with other speeds (low speed, *p* = 0.015*; medium speed, *p* = 0.003*; mixed speed, *p* = 0.008*); (3) Classifier employed had a marked effect on recognition accuracy. BiLSTM obtained significantly lower recognition accuracy than KNN (*p* = 0.021*) and SVM (*p* = 0.001*). Nonetheless, no significant difference (*p* = 0.236) was found between the recognition accuracy achieved using KNN and SVM.

**Table 3:** The results of multi-variable statistical analysis for crawling pattern recognition.

Factors		Sig. for Accuracy	Multiple comparisons (speed)		Sig. for accuracy
Main	Way	0.000**	Low	Medium	0.572
	Speed	0.011*		Fast	0.015*
	Classifier	0.002*		Mixed	0.832
			Medium	Fast	0.003*
				Mixed	0.724
			Fast	Mixed	0.008*

Multiple comparisons (way)		Sig. for accuracy	Multiple comparisons (classifier)		Sig. for accuracy
Participant-specific	Multi-Participant	0.938	KNN	BiLSTM	0.021*
	Participant-independent	0.000**		SVM	0.236
Multi-participant	Participant-independent	0.000**	BiLSTM	SVM	0.001*

(*p < 0.05, **p < 0.001).

### 3.2 Statistical results of stance duration, swing duration, and duty factor of stance phase

**Figure 10:**
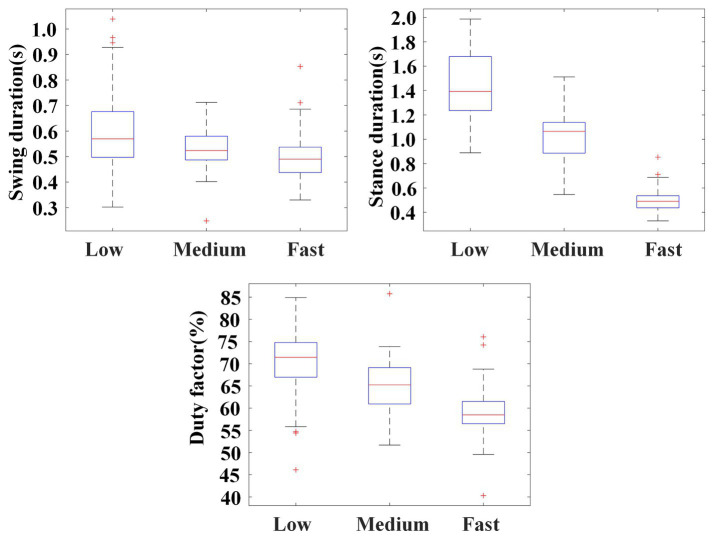
Illustration for swing durations, stance durations, and the duty factors of the stance phase of the left palm in self-selected crawling mode.

Figure 10 illustrates the statistical results of swing durations, stance durations, and the duty factors of the stance phase of the left palm. The results show that, as crawling speed increased, there was a substantial decrease in the duration of the stance phase (Low: 1.437 ± 0.255 s; Medium: 1.009 ± 0.220 s; High: 0.717 ± 0.135 s), while the duration of swing phase remained unchanged or slightly shortened (Low: 0.601 ± 0.149 s; Medium: 0.531 ± 0.078 s; High: 0.496 ± 0.089 s). On the whole, the duty factor of the stance phase decreased with the crawling speed (Low: 70.40 ± 6.54%; Medium: 64.97 ± 5.32%; High: 58.98 ± 4.81%).

### 3.3 Classification results of self-selected ILCM

**Figure 11:**
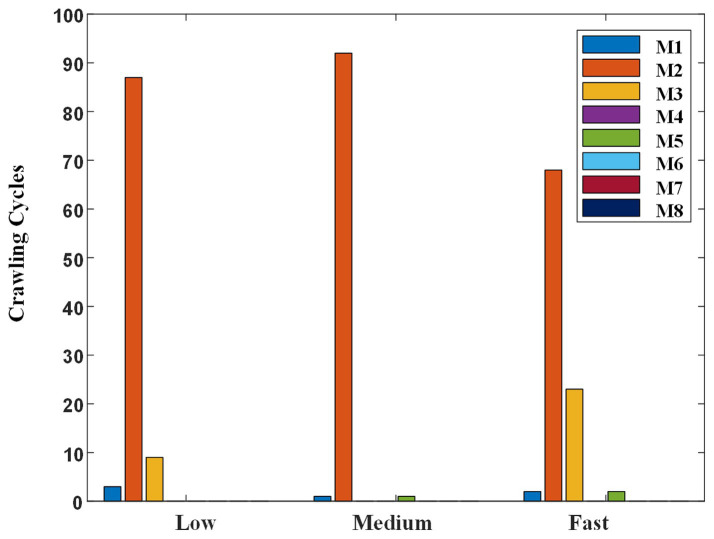
Classification result of self-selected crawling mode.

To figure out the possible reasons why the participants’ self-selected ILCM changes with crawling speed, the self-selected ILCM was classified using the KNN classifier, which was trained in the multi-participant way at mixed speed, and the results were shown in Figure 11.

Regardless of the crawling speed, M2 accounted for the largest proportion. At low, medium, and fast speeds, 87, 92, and 68 cycles were classified as M2, respectively. Meanwhile, the proportion of M3 changed with the crawling speed. Concretely, the crawling cycle number of M3 was 9, 0, and 23 at low, medium, and fast speeds, respectively. That is to say, when participants crawled at low speed or fast speed, although the trot gait M2 was the most adopted one, some participants preferred to choose M3 instead of M2, especially at fast speed. In addition, some ILCMs, such as M4, M6, M7, and M8, were adopted by nobody at any speed.

## 4 Discussion

### 4.1 The clinical application value of the EMG-based crawling pattern recognition scheme

Humans or quadruped animals have multiple ILCMs. Hildebrand divided the ILCMs of quadruped into two categories: symmetrical gaits and asymmetrical gaits (Hildebrand, 1976). Furthermore, symmetrical gaits were divided into four ILCMs in general: pace, lateral sequence, trot, and diagonal sequence (Hildebrand, 1976). Owaki et al. concluded that there were nine ILCMs in quadruped animals, i.e., lateral-sequence walk, diagonal-sequence walk, trot, pace, pronk, canter, bound, transverse gallop, and rotary gallop (Owaki and Ishiguro, 2017). Bellardita et al. found that wild-type mice had four ILCMs, namely, walk, trot, bound, and gallop (Bellardita and Kiehn, 2015). As for human crawling motion, IPL value has usually been used to classify ILCM into pace, trot, and no-limb-pairing modes in most studies adopting observation method or kinetics-based sensing technologies (Patrick et al., 2009, 2012; MacLellan et al., 2012, 2013; Righetti et al., 2015; Chen et al., 2017; Ma et al., 2017; Vitali et al., 2019). However, when ILCMs were simply considered as these three modes, many details of human crawling motion were ignored.

Unlike relevant research studies, eight ILCMs were defined in this study, and the EMG-based pattern recognition scheme was first introduced into the classification of crawling ILCMs. The experimental results demonstrated that the eight defined ILCMs could be distinguished with relatively high accuracies at different crawling speeds using three classifiers. Additionally, the classification performance of the EMG-based crawling pattern recognition scheme is very robust and less affected by crawling speed. Even in the participant-independent way, the SVM classifier achieves above 98% recognition accuracy at low, medium, and mixed speeds. In contrast to the rough classification based on IPL value, the EMG-based pattern recognition scheme could classify more detailed ILCMs during human crawling. As shown in Table 1, M2, M3, and M8 were usually classified into the trot mode according to IPL value; however, as shown in Figure 9, these three modes can be completely distinguished by utilizing the EMG-based pattern recognition scheme. The research results in this study verify the feasibility of providing an EMG-based crawling motion analysis method for clinical doctors. Specifically, we believe that the EMG-based pattern recognition scheme has potential application value in the fields of crawling function evaluation, understanding abnormal crawling control mechanisms and designing rehabilitation robots.

#### 4.1.1 Crawling ability assessment

Crawling is an important component of the Gross Motor Function Classification System (GMFCS) for children with cerebral palsy (Palisano et al., 2000). For example, for children under 2 years of age, children classified as GMFCS Level I should be able to crawl, stand up, grab furniture, and take a few steps, while children classified as GMFCS Level II can crawl on all fours or on both hands and feet. However, the evaluation based on subjective judgment lacks objectivity when clinicians use the GMFCS scale in the clinic. Instead, the proposed EMG-based pattern recognition scheme can provide a relatively objective evaluation of the crawling function of patients with cerebral palsy, stroke, or muscle atrophy. For instance, the patients could be asked to crawl using one or several ILCMs defined in this study, and the accuracy of completing specific ILCM modes can be obtained through the EMG-based pattern recognition scheme and serve as a basis for evaluating the patients’ crawling function.

#### 4.1.2 Understanding abnormal crawling control mechanisms

The EMG-based crawling pattern recognition scheme can help clinicians understand the abnormal crawling motion control mechanism of patients. The neuromuscular control mechanism of the central nervous system can be explained by the muscle synergy theory, which holds that when completing a specific motion, muscles are recruited and activated in groups rather than individually. By using the blind source separation algorithm to decompose the electromyographic signal envelope data, the synergy structure matrix that represents the group activation of muscles and the synergy recruitment curve matrix that represents the moment when the synergy structure matrix is recruited can be obtained. In our previous study (Li et al., 2023), the common synergy structure and common recruitment curve characteristics of 10 healthy adults when crawling on their hands and knees to complete different ILCMs were depicted. It was discovered that the CNS achieves crawling modes M3 ~ M8 by recruiting four inter-mode shared synergy structures associated with the swing function of each limb and achieves crawling modes M1 ~ M2 by synchronously recruiting two intra-mode shared synergy structures. Using similar research ideas, it is possible to obtain the common synergy structure and common synergy recruitment curve characteristics of healthy children. Clinicians can evaluate abnormal neuromuscular activation in children with cerebral palsy by comparing the similarities and differences in the synergy structure and synergy recruitment curves of children with cerebral palsy and the common synergy structure and common synergy recruitment curves of healthy children of the same age under the same ILCM. However, it is difficult to accurately label the ILCMs of multiple consecutive crawling cycles of children with cerebral palsy and their electromyographic signals through conventional observation and recording. Using the pattern recognition scheme proposed in this study, the crawling data of children with cerebral palsy can be more easily labeled to facilitate subsequent muscle synergy analysis.

#### 4.1.3 Rehabilitation robots design

Although crawling machines for physical exercise in healthy adults have been manufactured by FITCRAWL, an Australian company (FitCrawl, 2023), research on crawling robots for rehabilitation training of motor dysfunction was still in its early stages. Ghazi et al. designed an assistive crawling device to assist children with cerebral palsy in acquiring crawling skills. They mentioned their utilization of EEG-based neuro-imaging and a self-developed wearable motion-capture system (kinematic suit) alongside traditional methods for monitoring infant development (Ghazi et al., 2016). EMG signals not only carry motion control information from CNS such as EEG signals but also exhibit excellent performance in motion intention recognition, especially fine finger movement recognition (Chen et al., 2020). Therefore, we believe that the EMG-based crawling pattern recognition scheme has the potential to be applied in rehabilitation robot design to assist patients during crawling rehabilitation training with different inter-limb coordination modes.

### 4.2 The exploration of why humans choose various ILCMs at different crawling speeds

Researchers have conducted extensive studies on ILCMs of crawling motion. Freedland and colleagues observed that after 2 weeks of crawling on hands-knees, a convergence in inter-limb coordination among six infants toward a trot gait (Freedland and Bertenthal, 1994). Patrick et al. (2012) noted that all 22 infants demonstrated organized and rhythmic inter-limb coordination, with a predominance of trot gait and no instances of pace gait observed. Chen et al. (2017) claimed that participants utilized no-limb-pairing gait at low-speed crawling but switched to pace or trot gait at high speed. McElroy et al. (2008) declared that lizards preferred to adopt trot gait at high speeds. Although trot gait was a commonly adopted crawling gait, other gaits were also reported in the literature. In the report of Cole et al., trot gait was predominant not only during crawling on hands-knees but also during crawling on hands-feet. They also observed other gaits such as pace, single foot, gallop, and bound (Cole et al., 2019). Righetti et al. (2015) asserted that human infants would like to choose the lateral sequence walking gait (left knee, left palm, right knee, and right palm). Hildebrand’s research indicated that trot gait was more common in short-legged animals, while pace gait was more prevalent in long-legged animals (Hildebrand, 1976). In addition, diagonal sequence gait was found to generally appear in primates (Shapiro and Raichlen, 2006), and lateral sequence gait generally appeared in non-primate quadrupeds (Hildebrand, 1965, 1967; Vilensky, 1987).

Owaki et al. (2013) thought that trot coordination was the most energy-efficient gait for quadruped robots and had great potential for the design of controllers for quadruped robots (Owaki and Ishiguro, 2017). Their viewpoint can, to some extent, explain the reason why most humans and quadrupeds choose contralateral gait. As for the eight ILCMs defined in this study, their energy-saving efficiency can be analyzed using the central pattern generators (CPGs) model. CPG circuits are well known for their efficiency by requiring less reliance on higher brain center commands once activated. In our previous study (Li et al., 2023), a two-level CPG model was utilized to elucidate the role of CPG in facilitating cyclic locomotion during crawling. The model consists of a half-center rhythm generator (RG), which can be represented by the synergistic recruitment curve, and a pattern formation (PF) circuit, which can be represented by the synergistic structure. The RG facilitated the recruitment of synergies associated with the swing phase, enabling the control of a specific limb. In crawling modes M3 ~ M8, the crawling task was accomplished by sequentially recruiting four limbs, while crawling modes M1 and M2 involved the simultaneous recruitment of two limbs. Therefore, M1 and M2 seemed to be more energy-efficient than M3 ~ M8.

In this study, the classification results of the self-selected crawling mode showed that trot gait (M2) was the most adopted, which was consistent with previous studies. Meanwhile, the ILCM chosen by the participants has been found to change with crawling speed. As shown in Figure 11, when participants crawled at medium speed, there were no crawling cycles classified into M3. However, when participants crawled at low speed or fast speed, the proportion of M3 increased. Especially when participants crawled at a fast speed, M3 accounted for 24.2%. According to the definitions, the landing sequence of the limbs for M2 was left palm, right knee→ right palm, left knee→ left palm, right knee and the landing sequence of the limbs for M3 was left palm→ right palm→ left knee→ right knee→ left palm. Therefore, the main difference between trot gait M2 and diagonal sequence gait M3 was whether the left palm and right knee or right palm and left knee landed simultaneously or separately. In other words, M3 worked in a three-limb support mode, while M2 worked in a two-limb support mode. In theory, the three-limb support mode can offer higher stability than the two-limb support mode. That is to mean that M3 may provide higher stability than M2. On the other hand, as shown in Figure 10, the statistical results of crawling cycles demonstrated that humans mainly achieved an increase in crawling speed by reducing the duty factor of the stance phase. According to common sense, the longer the support time, the more stable the body was. Therefore, increasing crawling speed leads to a decrease in the duty factor of the stance phase, which can result in reduced physical stability.

Based on the above analysis, the reasons why humans chose various ILCMs at different crawling speeds can be explained from the perspectives of energy consumption and body stability. First, as the most energy-efficient gait, trot mode accounted for the vast majority at various speeds. Specifically, the proportion of trot gait at medium speed was the highest, at 92.9%. Therefore, energy conservation should be the main consideration when humans crawl; Second, at fast crawling speed, although energy consumption was still the main consideration, some participants who were more concerned about physical stability will choose the more stable crawling mode M3 instead of M2. As for the presence of a small amount of M3 gait at low speed, it should be the free choice made by the participants without considering energy conservation and physical stability.

### 4.3 Limitations

First, the design of the classifiers, such as BiLSTM, KNN, and SVM, was relatively simple, and there was a need to explore more innovative pattern recognition algorithms in the future; Second, only hands-knees crawling was targeted in this study, and future research should focus on more crawling postures; Third, it was necessary to recruit participants from different age groups to further validate the feasibility of the proposed EMG-based crawling pattern recognition scheme; Fourth, when applying the proposed scheme to clinical analysis, the data acquisition system should meet the requirements of miniaturization and wearability. A more feasible solution is to change the data transmission method from the existing wired transmission to a wireless transmission. Additionally, in clinical practice, collecting up to 30 channels of EMG data from patients will be a time-consuming and labor-intensive task. How to ensure that the recognition rate does not drop significantly while reducing the number of EMG electrodes as many as possible needs to be explored.

## 5 Conclusion

This study first introduced the EMG-based motion intention recognition technology into the classification of ILCMs during human crawling. Through experiments using different classifiers to classify the eight defined ILCMs at different speeds, it was verified that the EMG-based pattern recognition schemes can provide a more detailed classification of ILCMs, thereby confirming the feasibility of providing an EMG-based crawling motion analysis technology for clinicians. Furthermore, based on the classification results of self-selected crawling mode and the statistical results of stance duration, swing duration, and duty factor of stance duration, the exploration of why humans chose various ILCMs at different crawling speeds was approached from perspectives including energy consumption and body stability. The research results of this study have the potential application value for crawling function evaluation, understanding abnormal crawling control mechanisms, and designing rehabilitation robots.

## Data availability statement

The original contributions presented in the study are included in the article/supplementary material, further inquiries can be directed to the corresponding author.

## Ethics statement

The studies involving humans were approved by the Ethics Review Committee of Anhui Medical University (No. PJ 2014-08-04). The studies were conducted in accordance with the local legislation and institutional requirements. Written informed consent for participation in this study was provided by the participants’ legal guardians/next of kin. Written informed consent was obtained from the individual(s) for the publication of any potentially identifiable images or data included in this article.

## Author contributions

CL: Data curation, Formal analysis, Investigation, Methodology, Resources, Software, Visualization, Writing – original draft, Writing – review & editing. XC: Formal analysis, Funding acquisition, Supervision, Writing – original draft. XZ: Conceptualization, Software, Visualization, Writing – original draft. DW: Data curation, Writing – original draft.
